# Balancing Surgical Innovation and Risk: A Narrative Review of Emerging Technologies, Regulation, and Global Access

**DOI:** 10.7759/cureus.87957

**Published:** 2025-07-14

**Authors:** Sarup Saroha, Akash Patel

**Affiliations:** 1 General Surgery, University College London, London, GBR; 2 Trauma & Orthopaedics, Royal Free Hospital, London, GBR

**Keywords:** ai in healthcare, frugal innovation, global health equity, patient safety, regulatory frameworks, surgical innovation, virtual reality in surgery

## Abstract

Surgical innovation has significantly advanced patient care, enabling complex procedures and improving outcomes. However, the integration of new technologies presents challenges related to patient safety, regulatory oversight, cost-effectiveness, and equitable global access. The balance between fostering innovation and ensuring responsible implementation remains a critical issue in modern healthcare.

This narrative review explores the evolution of surgical innovation, highlighting key historical advancements, regulatory frameworks, and emerging technologies such as artificial intelligence (AI) in intraoperative decision-making and virtual reality (VR) in surgical education, as illustrative examples of transformative technologies. It discusses financial and ethical considerations, including the impact of frugal innovation in low-resource settings and the role of tiered regulatory approaches in mitigating risk.

Effective adoption of surgical innovations requires adaptable regulatory models, continuous training programmes, and a multi-stakeholder approach that includes policymakers, healthcare providers, and industry leaders. Addressing financial constraints and ethical dilemmas is essential to ensure that novel surgical technologies are accessible, safe, and sustainable.

Future efforts should prioritise dynamic regulatory mechanisms, equitable innovation dissemination, and interdisciplinary collaboration to maximise the benefits of surgical advancements while minimising risks. A structured approach, balancing technological progress, patient safety, and global equity, is crucial to shaping the future of surgery.

## Introduction and background

Surgical innovation has been a driving force in improving patient outcomes, enabling complex procedures that were once deemed impossible [1]. However, integrating these innovations into clinical practice presents challenges, particularly in balancing patient safety with financial sustainability in publicly funded healthcare systems such as the NHS [2,3].

This review explores the role of robust regulatory frameworks and tiered approaches in managing surgical innovation, with a particular focus on patient safety. It also examines the ethical implications of these advancements, addresses global disparities in access to care, and highlights strategies to ensure that progress in surgery benefits all, while maintaining safety and sustainability.

Historical innovations and initial challenges

**Figure 1:**
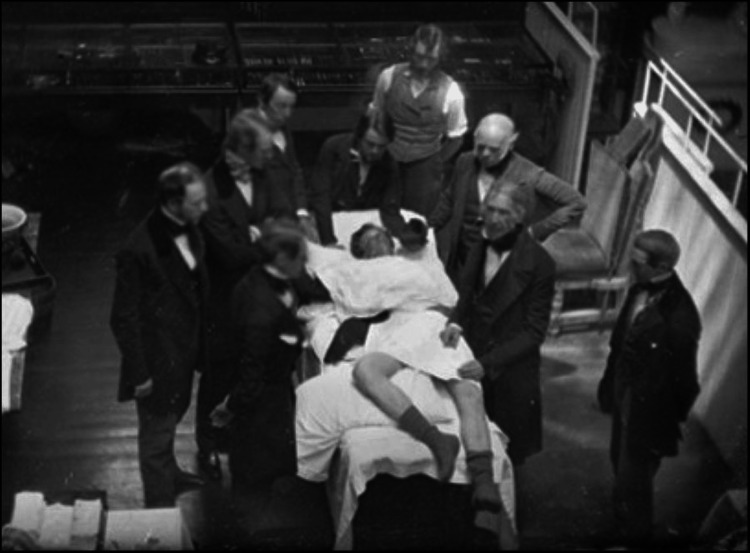
An early ether anaesthesia surgery, Massachusetts General Hospital, Boston, 1847. The use of anaesthesia marked a pivotal moment in surgical history, illustrating the challenges and transformative impact of integrating innovations into clinical practice. Used with permission from ref [8].

Understanding the historical trajectory of surgical innovation provides critical context for evaluating present-day challenges. Surgical practice has dramatically evolved over the past two centuries, with breakthroughs such as antiseptics, anaesthesia, and minimally invasive surgery transforming patient care (Figure 1). Incorporating these advancements into practice, however, has not been without challenges. Ethical concerns have frequently arisen [4-7], particularly regarding the lack of rigorous study design or regulatory oversight in some innovations. Despite these challenges, progress has marched forward, driven by a combination of imaginative vision and a structured, methodical approach.

**Figure 2:**
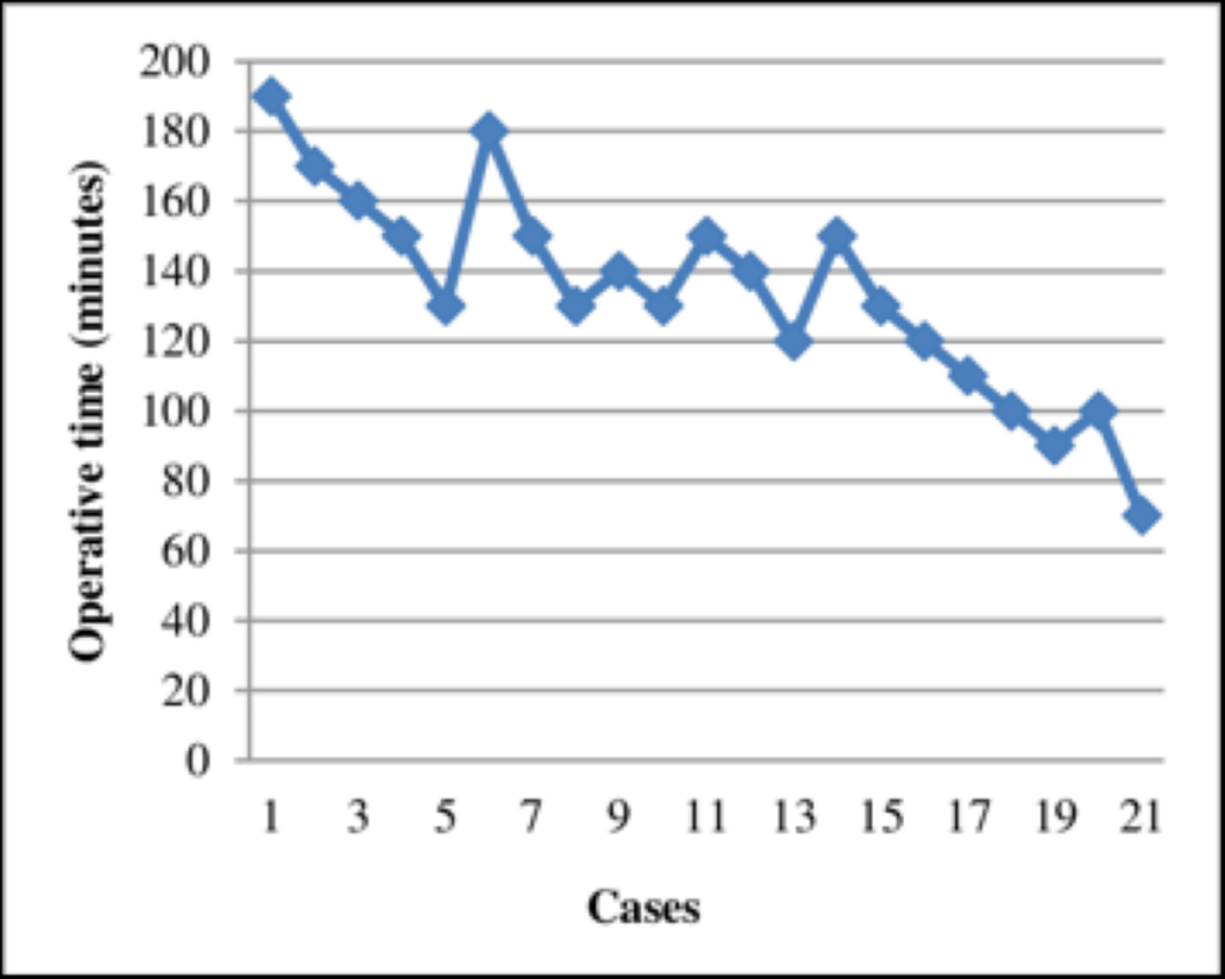
The learning curve effect in laparoscopic hysterectomy. This demonstrates the impact of surgical experience on operative times being reduced from ~3 h initially to ~1 h in the final cases. Image reproduced from ref [11], an open access article distributed under the terms of the Creative Commons Attribution License (CC BY).

For instance, when anaesthesia was introduced in the mid-19th century, it faced significant resistance. Many surgeons believed that pain was essential for the healing process [9], and this, coupled with concerns about other potential risks, overshadowed the clear benefits that anaesthesia offered. Today, this historical resistance highlights the importance of overcoming entrenched beliefs and carefully evaluating the evidence behind new practices, principles that continue to inform current surgical innovations. Similarly, the introduction of laparoscopic surgery in the 1990s, whilst transformative, presented a steep learning curve that initially led to an increase in surgical errors (Figure 2) [10,11]. This underscores the need for continuous evaluation and timely adjustment of training and regulatory frameworks to ensure that new techniques are safely integrated into clinical practice.

The evolution of regulatory frameworks

**Figure 3:**
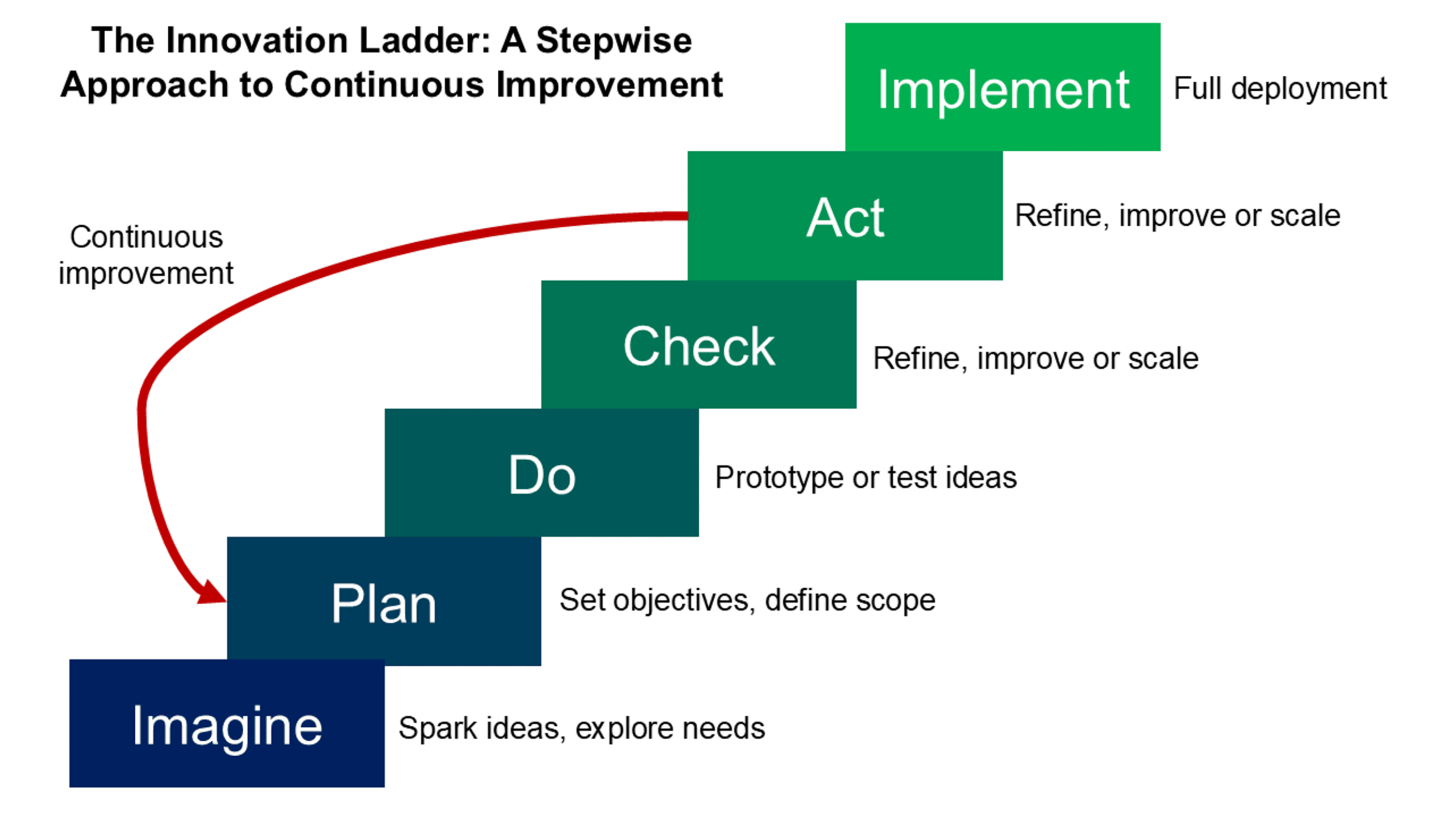
Innovation in surgery. From imagination to implementation, through a “plan, do, check, act” quality cycle.

The challenges faced during the adoption of laparoscopic surgery are mirrored today, where emerging technologies such as robotic surgery and AI-assisted procedures require equally rigorous oversight and training protocols to mitigate potential risks. In the early days, surgical advancements were largely unregulated, with individual surgeons responsible for patient safety. The development of regulatory frameworks has been crucial in managing these innovations through the oversight of formal regulatory bodies, such as the Medicines and Healthcare Products Regulatory Agency (MHRA) in the UK, the Food and Drug Administration (FDA) in the USA, and the European Medicines Agency (EMA). Innovations are evaluated rigorously before implementation in widespread clinical practice, thereby reducing patient risks. This approach involves a deliberate, step-by-step process of thorough evaluation and feedback, closely resembling the continuous quality improvement cycle (Figure 3) [1].

## Review

While the development of regulatory frameworks has been crucial in managing surgical innovations, these frameworks are not without limitations. The rapid pace of technological advancement often outstrips the ability of regulatory bodies to adapt, leading to potential gaps in oversight. Furthermore, the lengthy approval processes for new surgical technologies can delay the introduction of beneficial innovations to patients [9]. Existing regulations may also not fully address the ethical complexities of modern technologies.

A pertinent example is the integration of virtual reality (VR) into surgical training. VR exemplifies how new technologies can overcome challenges associated with traditional methods, such as cadaver training, which is costly, limited in accessibility, and lacks pathological diversity [12,13]. VR offers a cost-effective and widely accessible alternative, enabling surgeons to practise and refine techniques in a risk-free environment, simulating a wide range of clinical scenarios, including complex pathologies. This innovation shortens the learning curve and enhances patient safety [14-16], but cannot fully replicate the tactile experience of real surgery. Simulation must be integrated into a broader educational framework that includes supervised clinical experience and structured assessment, to prevent overreliance on virtual environments and the development of false confidence among trainees [17].

As with any emerging technology, the use of VR, and innovations such as AI-assisted surgery, necessitates evolving regulatory frameworks that can address the ethical and practical complexities, particularly concerning accountability and patient consent. This highlights the need for adaptive regulations that keep pace with technological advancements whilst safeguarding patient welfare.

Financial and global implications of surgical innovation

Building on the challenges of regulatory oversight, the financial and global implications of surgical innovation present additional hurdles that must be managed carefully to ensure equitable access and sustainable healthcare systems.

In the NHS, where budgets are already stretched thin, adopting expensive modern technologies on a large scale presents significant challenges. Decisions about investing in innovations such as robotics, AI, and VR often involve complex cost-benefit analyses, considering not only the direct financial costs but also the broader impact on patient care and healthcare equity. These technologies promise to transform surgery by offering greater precision, shorter recovery times, and improved patient outcomes [19]. However, substantial initial setup costs, additional training, and ongoing maintenance expenses hinder their integration into routine practice [20,21].

Compounding this issue is the tendency of NHS policymakers to prioritise immediate healthcare needs over preventive strategies that could reduce long-term costs and improve outcomes [22-24]. This reactive approach restricts significant upfront investments in advanced technologies, even when they have the potential to deliver considerable long-term savings.

These challenges are magnified further when considering the global context. In developed countries, advanced surgical technologies are generally more available, supported by robust healthcare systems capable of absorbing their substantial costs. However, introducing these technologies presents a far greater challenge in developing countries, where resources are limited.

A 2015 study revealed that five billion people lack access to safe, affordable surgical and anaesthesia care when needed [25]. This issue is most severe in low-income and lower-middle-income countries, where nine out of ten people are unable to obtain even basic surgical care [24,25].

This disparity raises critical ethical questions about the global distribution of healthcare resources and the responsibility of wealthier nations and international aid in supporting the adoption of life-saving surgical innovations in low-resource settings.

Frugal innovation [24], which designs or repurposes technologies for affordability in low-resource settings, offers a promising solution. For example, the Arbutus drill cover system [26], which can be attached to commercially available drills, was developed to improve access to safe surgery in resource-constrained environments.

Similarly, alternatives such as using paper clips instead of Raney clips in dental surgery [27], and sterilised mosquito net meshes as substitutes for commercial synthetic mesh in hernia repairs [28], exemplify how high-quality care can be provided at a fraction of the cost. These innovations demonstrate that advanced surgical techniques can be made accessible on a global scale, even in low-resource settings.

To manage the financial and global implications of surgical innovation effectively, society must adopt a collaborative approach. This involves healthcare providers, regulators, policymakers, and international organisations working synergistically to ensure that the benefits of surgical innovation are shared equitably across the globe. By adopting frugal innovation techniques designed specifically for low-resource settings, without compromising patient safety, we can achieve a more equitable distribution of healthcare resources.

Ultimately, the success of surgical innovation should be measured not only by its technical achievements but also by its capacity to bridge gaps in healthcare access and deliver life-saving care to those most in need.

Ethical considerations

Surgeons are the primary drivers of innovation in clinical practice, leading the implementation of new technologies and techniques. Whilst many of these innovations have significantly reduced patient morbidity and mortality, and improved outcomes, not all have been successful [29]. The inherent nature of innovation means that there may be a delay in recognising associated risks, sometimes not until thousands of patients have been studied. This uncertainty presents significant challenges for surgeons, patients, and the healthcare system.

Surgeons, therefore, bear a dual responsibility: to push the boundaries of knowledge whilst safeguarding patient safety and welfare. This requires a case-specific approach alongside regular audit of patient outcomes to continually refine the innovation. Additionally, surgeons must be mindful of the broader implications of their innovations, including impacts on healthcare costs and resource allocation.

A reflective, ethically grounded approach is essential to ensure that the benefits of surgical innovation are shared equitably, leaving no patient behind in the pursuit of medical progress. An ethical framework should be patient-specific and actionable, guided by the four fundamental medical ethics principles: autonomy, beneficence, non-maleficence, and justice (Table 1) [30,31].

**Table 1:** Key considerations for the ethical implementation of new surgical technologies and techniques, structured around the four principles of biomedical ethics: autonomy, beneficence, non-maleficence, and justice. These principles provide a foundation for evaluating innovative surgical practices in a way that balances patient safety, informed consent, equitable access, and clinical progress [30,31].

Key Questions
How is the safety of a new technology or technique guaranteed?
What is the process and timeline for implementing a new technology or technique in a hospital?
How are patients informed prior to undergoing a procedure involving a new technology or technique?
How are surgeons trained and certified in the use of a new technology or technique?
How are the outcomes of a new technology or technique monitored and assessed?
How are the responsibilities to individual patients balanced with those to society as a whole?

Autonomy

Autonomy centres on the patient’s right to make informed decisions about their care. As modern technologies are introduced into surgical practice, it is essential to ensure that patients fully understand the risks and benefits of these innovations. This becomes even more critical when dealing with experimental or unproven techniques, where the potential for unforeseen complications may be higher, and the long-term outcomes less clear. Informed consent must be rigorous, providing patients with clear, transparent information about potential outcomes, uncertainties, and alternatives. This process must go beyond formality, fostering a collaborative environment where patients are empowered to make truly informed decisions in partnership with their physicians. Tensions may arise between autonomy and the principles of beneficence and non-maleficence. Surgeons must respect patients' choices whilst ensuring those decisions are in the patient's best interest and do not cause harm. This conflict becomes pronounced when patients opt for risky, innovative procedures. The complexity of new technologies can make it difficult for patients to fully understand all implications, potentially leading to uninformed decisions. Surgeons must balance respecting autonomy with their ethical duty to guide patients toward safe and effective treatments.

Beneficence

Beneficence requires the promotion of patient well-being through the continual advancement of surgical care. Surgeons must strive to adopt innovations that not only improve clinical outcomes but also enhance the overall patient experience. This involves embracing new techniques and technologies that offer greater precision, faster recovery times, and better long-term results. Surgeons are entrusted with the responsibility to ensure that their pursuit of progress genuinely benefits patients, both in the short and long term. The pursuit of beneficence can, however, be in tension with non-maleficence and justice. For example, whilst an innovative technique may offer significant benefits to some patients, it might also carry risks that are not yet fully understood, potentially conflicting with the principle of "do no harm." Additionally, the high cost and limited availability of certain technologies can create disparities in access, challenging the principle of justice. Surgeons must navigate these tensions by carefully weighing the potential benefits of innovation against the risks and ensuring that advancements do not disproportionately favour certain patient groups over others.

Non-maleficence

Non-maleficence, the principle of "do no harm," demands that surgeons carefully balance the potential risks and benefits of new surgical technologies and techniques. As these innovations are developed, it is imperative that they are critically evaluated to prevent any potential harm to patients. This principle demands that all risks be minimised through meticulous planning, training, and ongoing assessment.

Justice

The principle of justice often conflicts with beneficence and autonomy in the context of surgical innovation. Whilst introducing a new technology may significantly improve outcomes, its high cost can limit availability, leading to inequities in access. Justice demands that healthcare resources, including innovative surgical technologies, be distributed equitably. However, the introduction of these technologies frequently exacerbates existing disparities, with wealthier patients or those in more developed regions benefiting disproportionately, whilst others are left behind [31]. This imbalance places a heavy responsibility on governments and healthcare providers to ensure that all patients, regardless of socioeconomic status, location, or other characteristics, have access to high-quality care. The uneven distribution of surgical innovations has tangible consequences, as it deepens social and economic inequalities. These global disparities highlight the urgent need for a more equitable approach to the dissemination of technology, particularly in underserved areas [32].

Balancing act: proposals for improvement

To effectively manage surgical innovation, it is essential to balance fostering technological advancements with ensuring patient safety, equity, and financial sustainability. The following four key proposals aim to address these challenges:

Tiered Regulatory Frameworks

A tiered regulatory framework categorises medical technologies based on their risk level, allowing for more streamlined approval of low-risk innovations whilst subjecting high-risk technologies to thorough evaluation (Table 2). Whilst this approach is used already by the United States Food and Drug Administration (USFDA) [33], the UK’s MHRA [34], and the EMA [35], it faces challenges in keeping pace with rapidly advancing technologies like AI and robotics, which introduce unique risks that current systems may not fully address.

**Table 2:** Demonstration of the concept of tiered regulatory frameworks in the context of surgical innovation Data taken from refs [33-35].

Regulation Tier Risk	Examples	Regulatory Oversight	Post-market Surveillance
Low	Non-invasive imaging tools (e.g., enhanced ultrasound software); surgical instrument design modifications (e.g., ergonomic adjustments to existing tools); improved surgical lighting systems	Streamlined approval process focusing on basic safety and efficacy checks. Minimal oversight with expedited pathways for low-risk modifications.	Basic monitoring of adverse events or device failures, often relying upon voluntary reporting by practitioners.
Medium	New surgical stapling devices; advanced laparoscopic instruments; AI-powered diagnostic tools for intraoperative decision-making (with proven safety in related applications)	Moderate evaluation involving targeted risk assessments specific to the innovation. Specific risk assessment protocols potentially involving limited clinical trials or pilot studies. Compliance with more stringent documentation and regulatory submission requirements compared to low-risk items.	Regular audits and reporting, including mandatory adverse event reporting and periodic reviews of performance data. Post-market studies may be required to confirm long-term safety and effectiveness.
High	New organ transplantation techniques; implantable Neurostimulation devices; novel prostheses	Thorough evaluation process, including comprehensive clinical trials to assess safety, efficacy, and potential long-term effects. Extensive clinical trials that might involve multi-phase testing, including preclinical studies, early human trials, and extensive post-approval trials. Detailed scrutiny by regulatory bodies often involving multiple rounds of review and expert consultation.	Continuous monitoring through registries or surveillance programs designed to track long-term outcomes and rare adverse effects. Detailed audits of both clinical and manufacturing data to ensure ongoing compliance. Regular updates to regulatory bodies with detailed reports on patient outcomes, adverse events, and product performance.

To enhance these frameworks further, integrating more dynamic and adaptive risk assessment processes is essential. For example, specialised review panels could be established for high-risk innovations, bringing together surgeons, patient advocates, and technology experts to ensure comprehensive oversight. Additionally, continuous dialogue between regulators, innovators, and healthcare providers is crucial for regularly updating risk criteria and optimising outcomes as new data and technologies emerge.

Continuous post-market surveillance is also vital, allowing for the prompt identification and correction of issues that arise after a technology's introduction. By refining these frameworks to be more responsive and inclusive, we can better balance the need for patient safety with the swift adoption of beneficial surgical innovations.

Continuous Education and Training

The rapid introduction of new surgical technologies necessitates continuous education and training for healthcare providers. Innovative training tools, such as VR, can shorten learning curves and enhance patient safety by allowing surgeons to practice in risk-free environments. Beyond technical skills, training should also incorporate the ethical implications of using new technologies, including informed consent and data privacy. Regulatory bodies and professional organisations should collaborate to develop standardised, regularly updated training curricula that address both the technical and ethical dimensions of surgical innovation. This approach would ensure that surgeons are equipped to use new technologies responsibly and effectively.

Focus on Frugal Innovation

Frugal innovation should be prioritised to democratise access to advanced surgical care in low-resource settings. Technologies that are cost-effective and maintain safety and efficacy can help bridge the gap between high-income and low-income healthcare systems. Governments and international organisations can play a pivotal role in supporting the development and dissemination of frugal innovations through targeted funding and incentives. However, financial support alone is not sufficient. Building local capacity to produce and maintain these technologies is essential for ensuring their long-term sustainability. Without local manufacturing capabilities, low-resource settings may remain dependent on external suppliers, exposing them to supply chain disruptions and increased costs over time. Additionally, global health organisations and governments should collaborate to create platforms for sharing best practices and successful models of frugal innovation, such as “innovation hubs” in partnership with industry and engineers [36]. This collaboration can accelerate the adoption of proven technologies across multiple regions, ensuring that the benefits of innovation are spread widely and equitably. By promoting frugal innovation, we can make advanced surgical care accessible to all, regardless of geographic or economic barriers.

Adaptive and Forward-Looking Regulatory Bodies

Regulatory bodies must adopt a proactive approach to managing emerging surgical technologies, moving beyond the traditional reactive model. They must not just respond to innovations as they emerge but must anticipate potential challenges before they become widespread to allow appropriate and timely intervention. Engaging with innovators early in the development process would allow regulators to guide technological advancements in ways that enhance patient safety without stifling innovation. A vital component of this approach is the development of forward-thinking guidelines that can adapt to the evolving landscape of surgical technologies. A practical example of this is the FDA’s Digital Health Software Precertification Program, which explores a streamlined, risk-based oversight model for AI-driven technologies by evaluating developers rather than individual products. This model reflects an effort to accommodate the iterative and adaptive nature of machine learning systems within a flexible regulatory framework [37]. As AI and robotics become more integrated into surgical practice, they introduce ethical and practical complexities that existing frameworks may not fully address. For example, AI systems raise questions about accountability, whilst robotic systems introduce risks related to machine error.

To address these complexities, regulatory bodies should collaborate closely with a broad range of stakeholders, including technologists, ethicists, patient advocacy groups, and surgical teams. Such collaboration would ensure that guidelines are comprehensive, considering both the technical safety of innovations and their broader societal impacts. Proactive regulation should also include mechanisms for continuous monitoring and feedback, allowing frameworks to evolve as technologies mature and their real-world applications become widespread. Finally, regulatory bodies should collaborate internationally, ensuring that surgical innovations with the potential to benefit humanity are not limited by national borders. Harmonising regulatory standards can prevent disparities in access to safe and effective technologies by ensuring that the benefits of innovation are shared equitably.

These four key proposals offer a balanced approach to managing surgical innovation, ensuring that technological advancements benefit all patients while maintaining safety, equity, and financial sustainability. By implementing these strategies, society can create a healthcare system that is both innovative and inclusive alike.

## Conclusions

Managing innovation in surgical practice requires balancing technological progress with ethical, financial, and global considerations. Whilst advances such as anaesthesia and laparoscopic surgery have transformed care, they also highlight the risks associated with inadequate oversight and training. As modern innovations like AI and robotics enter practice, rigorous and adaptive frameworks remain essential to protect patient autonomy and safety.

Additionally, global disparities in access to surgical innovation must be addressed. Frugal innovation offers a promising route to equitable care, particularly in low-resource settings. Ultimately, a proactive, inclusive approach to surgical innovation, guided by patient welfare, ethical principles, and sustainable investment, can ensure that progress benefits all, not just the privileged few.
